# Low-temperature fabrication of an HfO_2_ passivation layer for amorphous indium–gallium–zinc oxide thin film transistors using a solution process

**DOI:** 10.1038/s41598-017-16585-x

**Published:** 2017-11-24

**Authors:** Seonghwan Hong, Sung Pyo Park, Yeong-gyu Kim, Byung Ha Kang, Jae Won Na, Hyun Jae Kim

**Affiliations:** School of Electrical and Electronic Engineering, Yonsei University, 50 Yonsei-ro, Seodaemun-gu, Seoul, 03722 Republic of Korea

## Abstract

We report low-temperature solution processing of hafnium oxide (HfO_2_) passivation layers for amorphous indium–gallium–zinc oxide (a-IGZO) thin-film transistors (TFTs). At 150 °C, the hafnium chloride (HfCl_4_) precursor readily hydrolyzed in deionized (DI) water and transformed into an HfO_2_ film. The fabricated HfO_2_ passivation layer prevented any interaction between the back surface of an a-IGZO TFT and ambient gas. Moreover, diffused Hf^4+^ in the back-channel layer of the a-IGZO TFT reduced the oxygen vacancy, which is the origin of the electrical instability in a-IGZO TFTs. Consequently, the a-IGZO TFT with the HfO_2_ passivation layer exhibited improved stability, showing a decrease in the threshold voltage shift from 4.83 to 1.68 V under a positive bias stress test conducted over 10,000 s.

## Introduction

Amorphous oxide semiconductor (AOS)-based thin-film transistors (TFTs) are promising alternatives for conventional amorphous silicon-based TFTs because of their superior electrical characteristics, such as high field-effect mobility (μ_FET_), low off-current, and high transparency in the visible range^1–4^. However, AOS TFTs have a significant issue of inferior bias instability due to the adsorption/desorption of oxygen and water molecules in the channel layer^5,6^. Various passivation layers, such as SiO_2_, SiN_x_, and Al_2_O_3_, have been adopted to address this issue^6–9^. These inorganic materials are generally deposited by vacuum processes including plasma-enhanced chemical vapor deposition (PECVD), pulsed laser deposition (PLD), and sputtering. However, vacuum-based processes have the disadvantages of being complex and costly, and plasma damage on the back surface of a channel can lead to performance degradation of TFTs^8–10^.

Solution-processed passivation layers have been explored to overcome the limitations of vacuum processes. These layers have the advantages of being simple processes, and are inexpensive and do not use potentially damaging plasmas. Organic materials, such as poly(methyl methacrylate) (PMMA)^11,12^, polydimethylsiloxane (PDMS)^5^, and polyacrylate (PA)^6^ are commonly used to fabricate solution-processed passivation layers. These materials have been suggested as passivation layers for flexible electronics because they can be fabricated at low temperatures, i.e., below 150 °C. However, they are more permeable to gases compared with inorganic materials, and hence the instability issue of AOS TFTs caused by the interaction between a channel and the ambient atmosphere cannot be completely eliminated^9,10^. Solution-processed passivation layers using inorganic materials, such as Y_2_O_3_ and Al_2_O_3_, have been studied as an alternative to organic passivation layers^13–16^. Although they are much better gas barriers than organic passivation layers, they must be fabricated at high temperatures, i.e., above 250 °C, which limits their use with some flexible substrates.

In this study, a solution-processed hafnium oxide (HfO_2_) passivation layer was fabricated at low temperature (150 °C) using an aqueous solution of hafnium chloride (HfCl_4_) because strongly hydrated HfCl_4_ decomposes and transforms into HfO_2_ at lower temperature than anhydrous HfCl_4_. The electrical characteristics and stability of the indium–gallium–zinc oxide (a-IGZO) TFT with HfO_2_ passivation were compared with those of a-IGZO TFTs without passivation, and with the commonly used PMMA and Y_2_O_3_ passivation^11–15^. Thermogravimetric analysis and differential scanning calorimetry (TGA/DSC) and X-ray photoelectron spectroscopy (XPS) analysis of the HfO_2_ passivation layer verified the formation of the HfO_2_ passivation layer at 150 °C. The effect of HfO_2_ passivation was also demonstrated by comparing the XPS depth profile results for a-IGZO TFTs without passivation and with HfO_2_ passivation.

## Results

**Figure 1:**
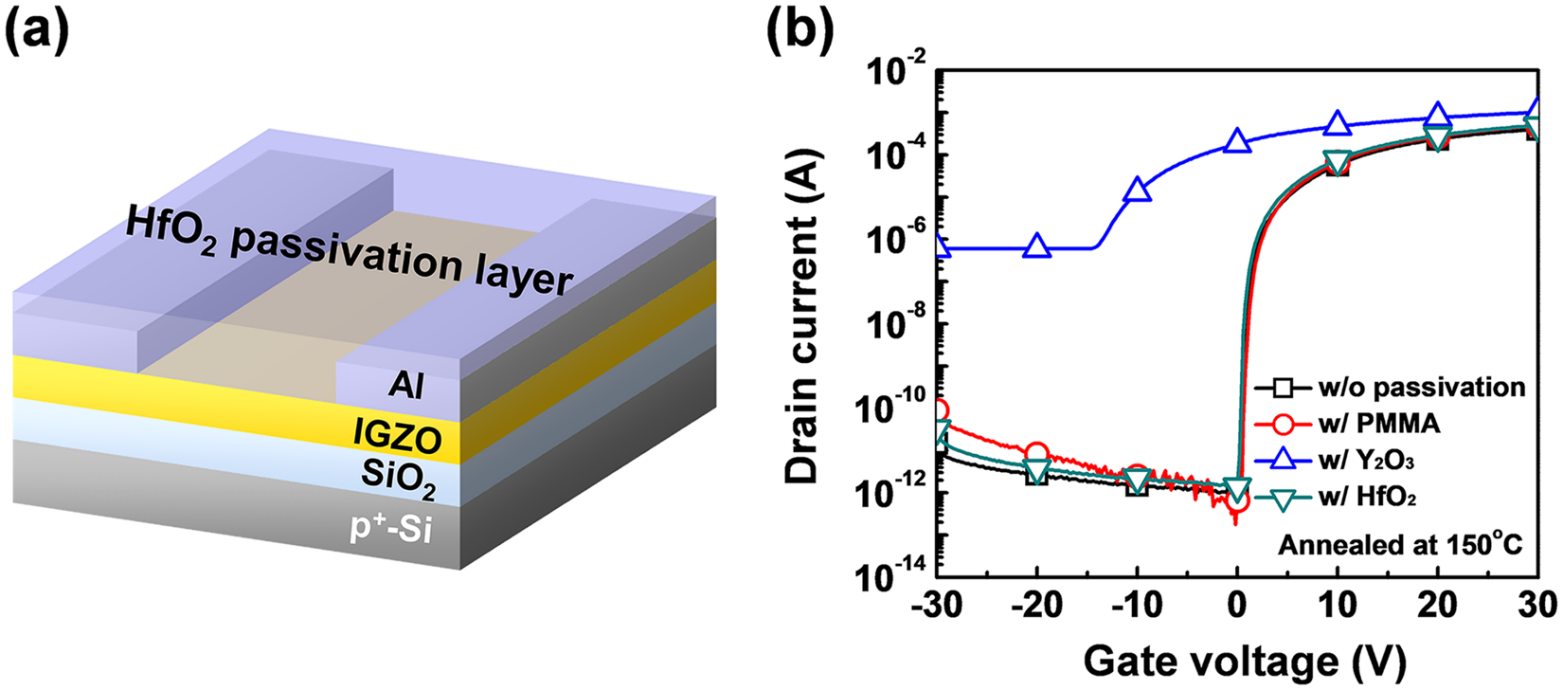
(**a**) Schematic structure of the a-IGZO TFT with solution-processed HfO_2_ passivation and (**b**) transfer characteristics of the a-IGZO TFTs without passivation and with PMMA, Y_2_O_3_, and HfO_2_ passivation annealed at 150 °C.

**Figure 2:**
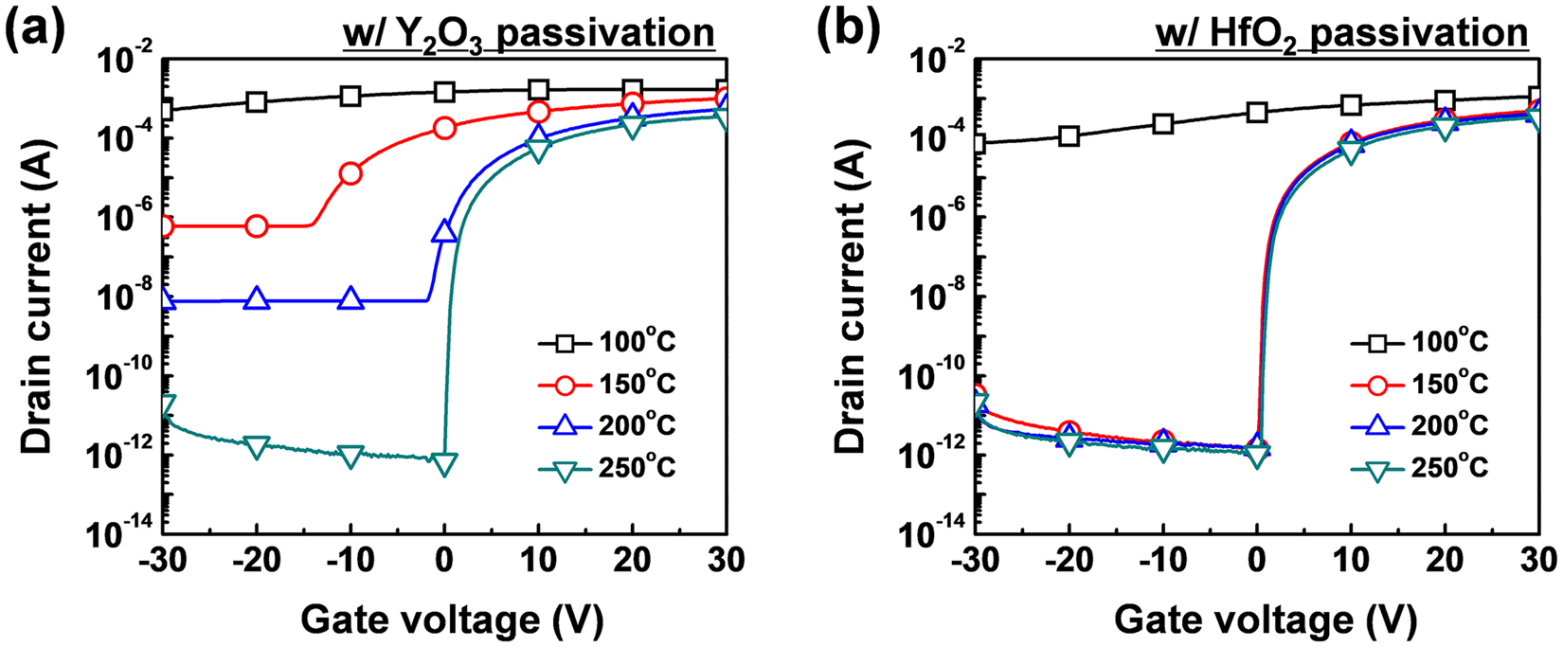
Transfer characteristics of the a-IGZO TFTs with (**a**) Y_2_O_3_ and (**b**) HfO_2_ passivation as a function of annealing temperature.

Figure 1(a) shows the deionized (DI) water-based solution process used to fabricate the a-IGZO TFTs with HfO_2_ passivation. The a-IGZO TFTs without passivation and with PMMA and Y_2_O_3_ passivation were also prepared for comparison. Figure 1(b) shows the transfer characteristics of the a-IGZO TFTs without passivation and with PMMA, Y_2_O_3_, and HfO_2_ passivation annealed at 150 °C. The 150 °C-annealed a-IGZO TFT with PMMA passivation, which is a commonly-used organic passivation layer, showed proper switching characteristic, as reported previously^11,12^. However, the a-IGZO TFT with Y_2_O_3_ passivation, which is the most widely studied solution-processed passivation among inorganic materials, showed no switching characteristic, while the a-IGZO TFT with HfO_2_ passivation showed proper switching characteristic^13–15^. This indicated that the thermal energy at the annealing temperature at 150 °C was insufficient for the Y_2_O_3_ precursor solution to form a passivation layer, which resulted in an excess carrier concentration in the channel layer^13^. The passivated a-IGZO TFTs were annealed from 100 to 250 °C to identify the minimum processing temperature for Y_2_O_3_ and HfO_2_ passivation. Figure 2(a) shows the evolution of the transfer characteristics for the a-IGZO TFT with Y_2_O_3_ passivation as a function of annealing temperature. It reveals that the annealing temperature of the solution-processed passivation layer made with the Y_2_O_3_ precursor solution should be ca. 250 °C to form a passivation layer. On the other hand, the HfO_2_ precursor solution would form an HfO_2_ passivation layer after thermal annealing at 150 °C. The transfer characteristic of the a-IGZO TFT with HfO_2_ passivation exhibited similar performance to that of the a-IGZO TFT without passivation (Fig. 2(b)). The μ_FET_, threshold voltage (V_th_), on/off ratio, and subthreshold swing (SS) of the a-IGZO TFT with HfO_2_ passivation were 9.60 ± 0.98 cm^2^/Vs, 1.49 ± 0.89 V, (2.54 ± 1.11) × 10^8^, and 0.35 ± 0.4 V/dec, respectively (Table 1).

**Table 1:** Extracted parameters of the a-IGZO TFTs without passivation and with PMMA, Y_2_O_3_, and HfO_2_ passivation annealed at 150 °C.

Condition	Mobility (cm^2^/Vs)	V_th_ (V)	On/off	SS (V/dec)
w/o passivation	9.26 ± 0.93	1.72 ± 0.56	(4.25 ± 0.68) × 10^8^	0.33 ± 0.3
w/PMMA	9.51 ± 0.86	1.68 ± 0.56	(8.54 ± 0.85) × 10^8^	0.32 ± 0.3
w/Y_2_O_3_	11.30 ± 1.35	−12.94 ± 1.98	(1.72 ± 6.44) × 10^3^	2.67 ± 0.6
w/HfO_2_	9.60 ± 0.98	1.49 ± 0.89	(2.54 ± 1.11) × 10^8^	0.35 ± 0.4

**Figure 3:**
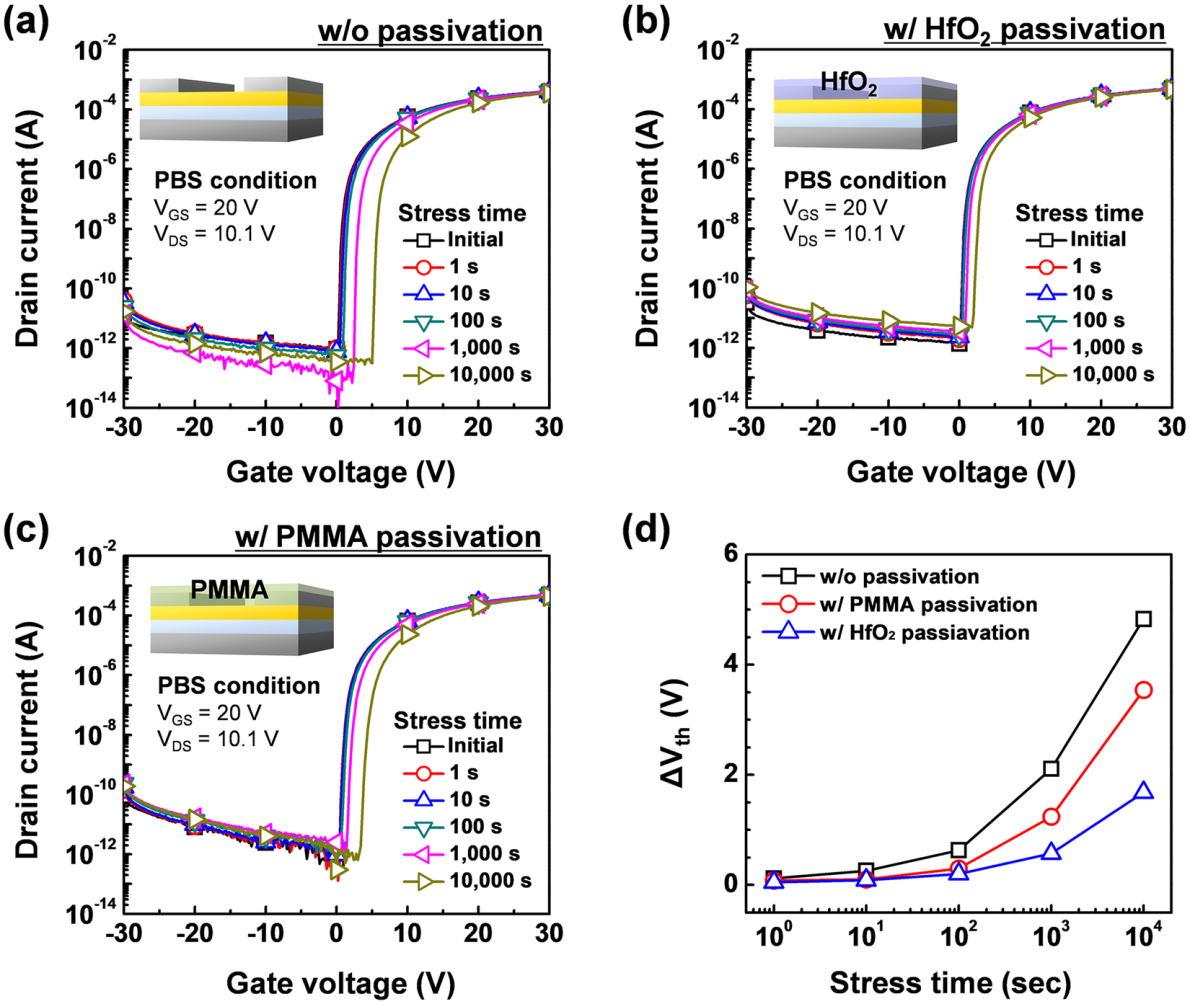
PBS test results of the a-IGZO TFTs (**a**) without passivation and with (**b**) PMMA and (**c**) HfO_2_ passivation, and (**d**) comparison of the PBS test results.

To confirm the effectiveness of the HfO_2_ passivation layer, the positive bias stress (PBS) test was performed for 10,000 s with V_GS_ = 20 V, and V_DS_ = 10.1 V. Figure 3(a,b) show the evolution of the transfer characteristics for the a-IGZO TFTs without passivation and with HfO_2_ passivation under PBS. After the test, the V_th_ shift (ΔV_th_) of the a-IGZO TFT with HfO_2_ passivation was 1.68 V, whereas that of the a-IGZO TFT without passivation was 4.83 V. Therefore, although the HfO_2_ passivation layer was fabricated at the low temperature of 150 °C, it was effective as a passivation layer. The a-IGZO TFT with PMMA passivation annealed at 150 °C was also subjected to the PBS test for comparison. The ΔV_th_ of the a-IGZO TFT with PMMA passivation was 3.54 V after 10,000 s, which was inferior to that of the a-IGZO TFT with HfO_2_ passivation (Fig. 3(c)). This demonstrated that the barrier property of the HfO_2_ passivation layer annealed at 150 °C was better than that of the PMMA passivation layer when processed at low temperature.

## Discussion

The thermal decomposition characteristics of the HfCl_4_ precursor for the HfO_2_ passivation layer depend on its hydration state. The HfCl_4_ starting material is weakly hydrated with a composition of HfCl_4_·1/6H_2_O. When using an anhydrous solvent for the HfO_2_ precursor solution, the HfCl_4_·1/6H_2_O decomposes into anhydrous HfOCl_2_ via the intermediate Hf(OH)Cl_3_, as follows:

1 $${{\rm{HfCl}}}_{4}+\frac{1}{6}{{\rm{H}}}_{2}{\rm{O}}\to \frac{1}{6}{\rm{Hf}}({\rm{OH}}){{\rm{Cl}}}_{3}+\frac{5}{6}{{\rm{HfCl}}}_{4}+\frac{1}{6}{\rm{HCl}}$$

2 $${\rm{Hf}}({\rm{OH}}){{\rm{Cl}}}_{3}\to {{\rm{HfOCl}}}_{2}+{\rm{HCl}}$$

Further reaction of the HfOCl_2_ leads to HfO_2_ and HfCl_4_, which sublimes at ca. 300 °C at atmospheric pressure^17,18^.

3 $$2{{\rm{HfOCl}}}_{2}\to {{\rm{HfO}}}_{2}+{{\rm{HfCl}}}_{4}$$

However, the hydrolysis reaction readily occurs when water is used as the solvent to form Hf(OH)_*x*_Cl_4−*x*_, as follows:

4 $${{\rm{HfCl}}}_{4}+x{{\rm{H}}}_{2}{\rm{O}}\,\to {\rm{Hf}}{({\rm{OH}})}_{x}{{\rm{Cl}}}_{4-x}+x\text{HCl}\,(x=1\,or\,2)$$

The Hf(OH)_*x*_Cl_4−*x*_ is unstable and transforms into HfOCl_2_, which leads to formation of the oxychloride octahydrate (HfOCl_2_·8H_2_O), as follows:

5 $${\rm{Hf}}({\rm{OH}}){{\rm{Cl}}}_{3}\to {{\rm{HfOCl}}}_{2}+{\rm{HCl}}$$

6 $${\rm{Hf}}{({\rm{OH}})}_{2}{{\rm{Cl}}}_{2}\to {{\rm{HfOCl}}}_{2}+{{\rm{H}}}_{2}{\rm{O}}$$

This strongly hydrated HfOCl_2_·8H_2_O has a tetrameric structure that has only doubly-bridging OH bonds. The HfOCl_2_ decomposes and transforms into HfO_2_ at ca. 150 °C according to the following reaction^17^:

7 $${{\rm{HfOCl}}}_{2}+n{{\rm{H}}}_{2}{\rm{O}}\,\to {{\rm{HfO}}}_{2}+2{\rm{HCl}}+(n-1){{\rm{H}}}_{2}{\rm{O}}$$

**Figure 4:**
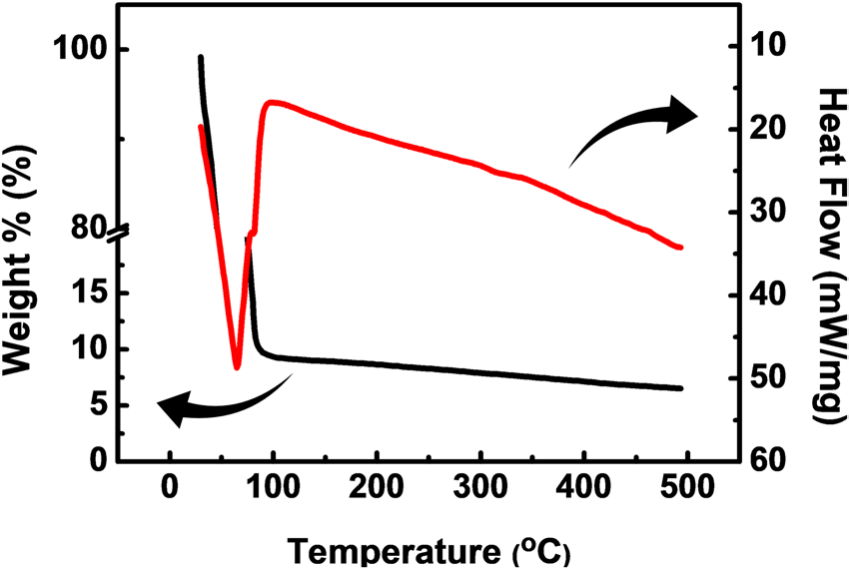
The TGA/DSC curves of the HfO_2_ precursor solution.

Figure 4 shows the TGA/DSC analysis result of the HfO_2_ precursor solution, where the HfCl_4_ is dissolved in DI water. There is an abrupt weight loss around 100 °C, but it is hard to distinguish the HfOCl_2_ decomposition from water solvent evaporation. This is due to the decomposition characteristic of strongly hydrated HfOCl_2_·8H_2_O. This result shows a distinguishable trend in decomposition temperature from the previously reported TGA result of anhydrous HfCl_4_ reported previously, where a large weight loss occurs between 200 and 300 °C^17^. Thus, the hydration state is the most significant parameter for the passivation temperature of solution-processed HfO_2_. Using water as the solvent is the best way to maximize the extent of hydration, which enables formation of the solution-processed inorganic passivation layer at low temperature.

**Figure 5:**
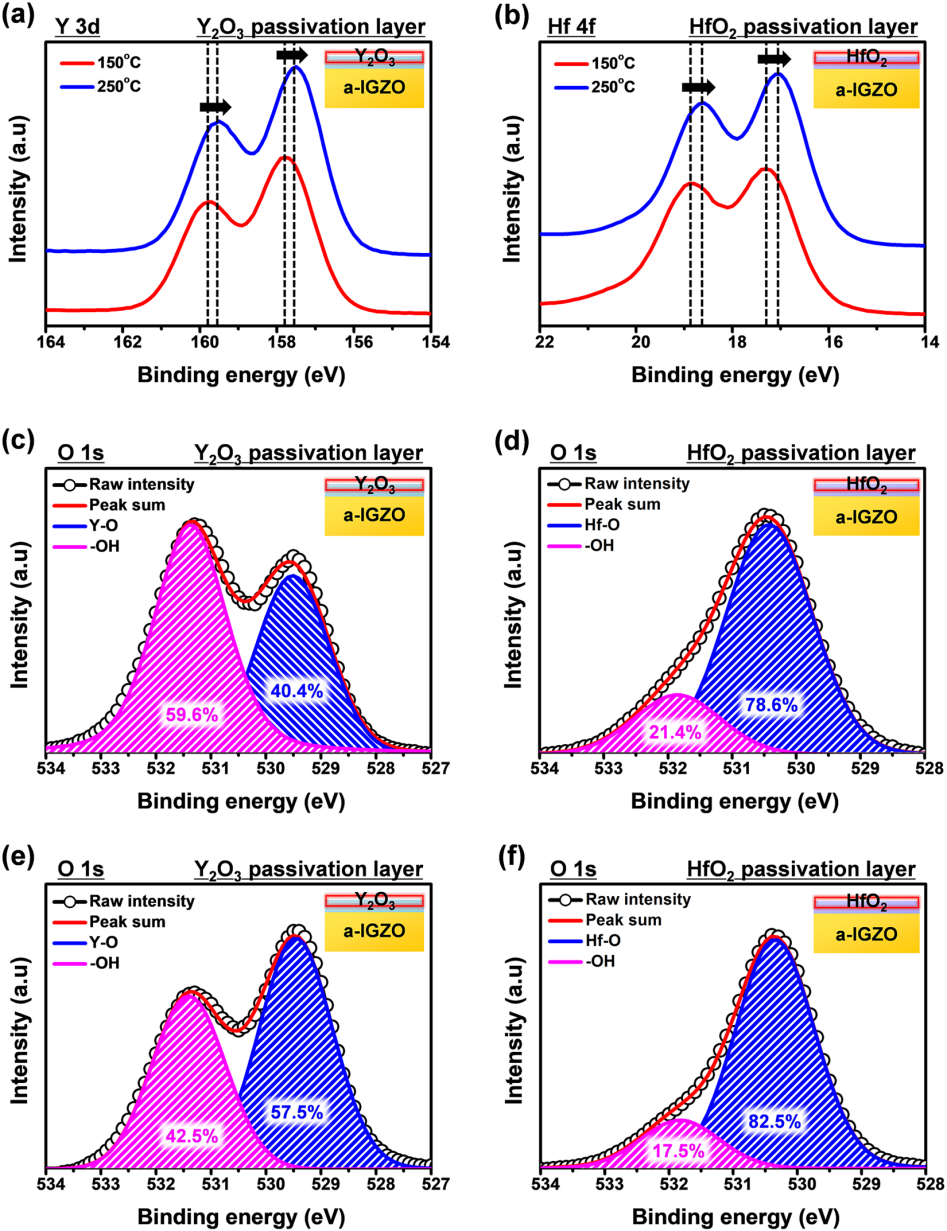
XPS results for (**a**) the Y 3d spectra of the Y_2_O_3_ and (**b**) the Hf 4 f spectra of the HfO_2_ passivation layer annealed at 150 °C and 250 °C, and the O 1s spectra of the (**c**) Y_2_O_3_ and (**b**) HfO_2_ passivation layer annealed at 150 °C, and the (**e**) Y_2_O_3_ and (**f**) HfO_2_ passivation layer annealed at 250 °C.

XPS was used to examine the solution-processed Y_2_O_3_ and HfO_2_ passivation layers that were annealed at 150 and 250 °C (Fig. 5). The Y 3d spectra of Y_2_O_3_ and Hf 4 f spectra of HfO_2_ showed doublet features (Fig. 5(a,b)). At the higher annealing temperature, the Y 3d_5/2_ and Y 3d_3/2_ peaks shifted from 157.8 to 157.5 eV, and from 159.7 to 159.5 eV, respectively, and the Hf 4f_7/2_ and Hf 4f_5/2_ peaks from 17.3 to 17.1 eV and 18.8 to 18.6 eV, respectively. This indicated that there was an increase in Y–O and Hf–O bonding, and a decrease in the number of hydroxyl groups at the higher annealing temperature^19–23^. Specific analyses for oxide and hydroxide were done by deconvoluting the O 1s spectra of the Y_2_O_3_ and HfO_2_ passivation layers, which had been annealed at 150 and 250 °C. The O 1s peak was deconvoluted into two peaks centered at 529.5 and 531.3 eV for Y_2_O_3_, and at 530.4 and 531.8 eV for HfO_2_ (Fig. 5(c–f)). The first peak corresponded to the binding energy of the oxide, and the second peak to the hydroxyl groups^18,19,24,25^. The O 1s spectrum of Y_2_O_3_ annealed at 150 °C was similar to that of the as-deposited film, indicating a large number (59.6%) of hydroxyl groups (Fig. 5(c) ^20^. This amount decreased to 42.5% as the annealing temperature increased to 250 °C (Fig. 5(e)), when the spectrum resembled that of a conventional Y_2_O_3_ film^20,21^. However, the O 1s spectrum of HfO_2_ was already similar to that of a standard HfO_2_ film when annealed at only 150 °C^18,19^. It had a small number of hydroxyl groups (21.4%), and there was a slight decrease for the 250 °C-annealed film. Therefore, the solution processed HfO_2_ passivation layer is sufficiently oxidized when annealed at 150 °C, and a small number of hydroxyl groups can ensure TFT reliability because dissociated hydrogen from hydroxide bonds can diffuse into a channel and affect the characteristic of TFT^26,27^.

In the PBS test, the principal origin of instability is the interaction between the ambient gas and the back surface of the TFT^6,8,28^. When a positive bias is applied to a gate, accumulated free electrons are captured by the adsorbed oxygen molecules on the back surface of a TFT. This can be mitigated with a passivating layer. Our results demonstrated that an effective HfO_2_ passivation layer could be formed at an annealing temperature of 150 °C, and this effectively reduced the interaction between ambient gases and the back-channel layer.

**Figure 6:**
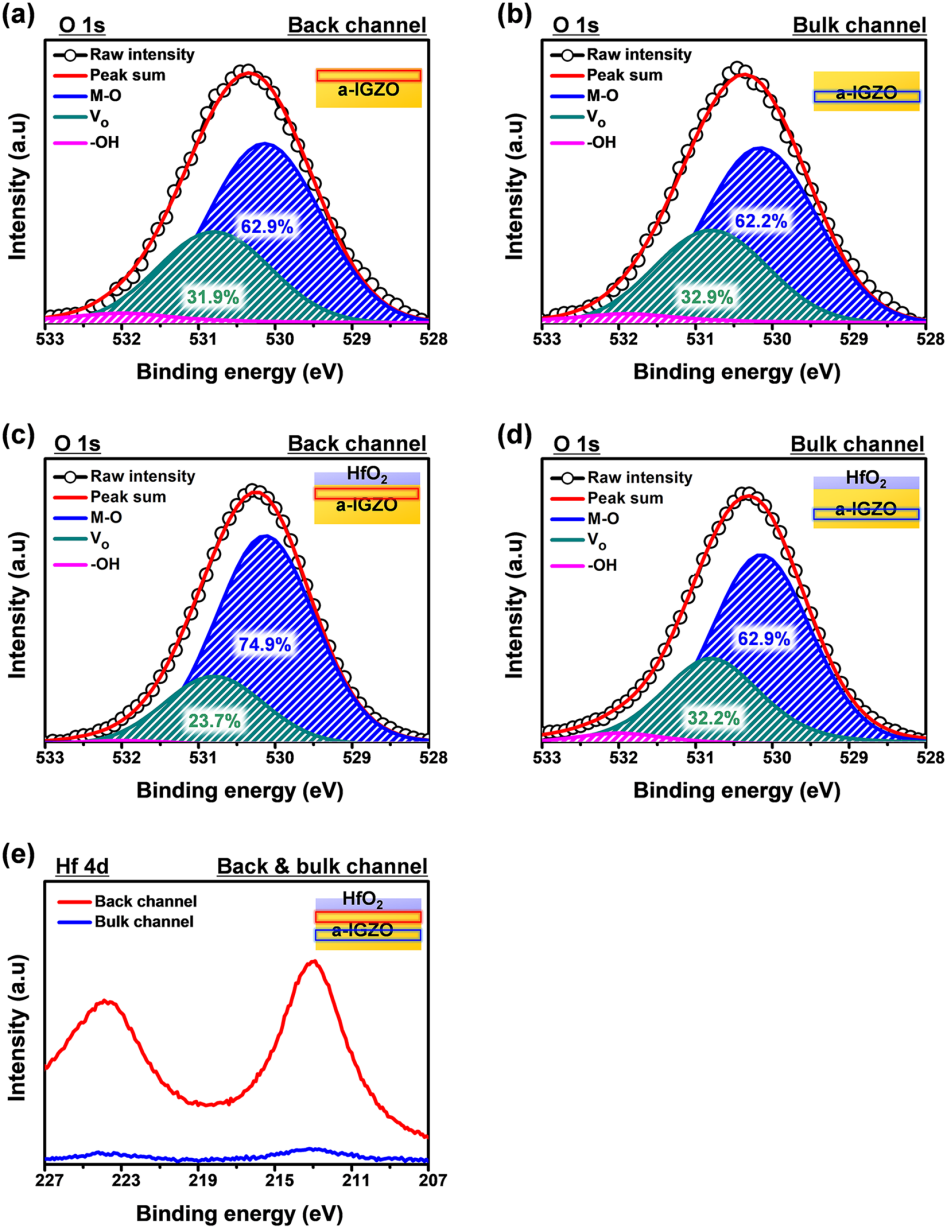
XPS depth analyses for the O 1s spectra of the (**a**) back- and (**b**) bulk-channel of the a-IGZO TFT without passivation, and the (**c**) back- and (**d**) bulk channel of the a-IGZO TFT with HfO_2_ passivation, and (**e**) the Hf 4d spectra of the a-IGZO TFT with HfO_2_ passivation.

The oxygen vacancy (V_o_) in the channel layer can act as a trap site and lead to PBS instability^15,29^. Hence, XPS depth analyses for the channel layers of the a-IGZO TFTs without passivation and with the HfO_2_ passivation layer were also made, to confirm additional benefits of the solution-processed HfO_2_ passivation. Figure 6(a,c) show the O 1s spectra for the back-channel region of the a-IGZO film without passivation and with HfO_2_ passivation, respectively, and Fig. 6(b,d) show the same for their bulk-channel regions. We used 25% of the total channel etching time of the a-IGZO film without passivation and with HfO_2_ passivation in the back-channel region that was adjacent to the back surface or HfO_2_ layer, and 75% of the total channel etching time in the bulk-channel region that is far from the back surface or HfO_2_ layer. The O 1s peak was deconvoluted into three peaks centered at 530.1 ± 0.2, 531.0 ± 0.2, and 532.0 ± 0.2 eV^29^. These features corresponded to In, Ga, and Zn metal oxide bonds (M–O), V_o_, and metal hydroxide species (–OH), respectively. For the a-IGZO film without passivation, there was little difference between the V_o_ ratios of the back- and bulk-channel regions; the V_o_ ratios for the back- and bulk-channel regions were 31.9 and 32.9% respectively (Fig. 6(a,b)). However, in the case of the a-IGZO film with HfO_2_ passivation, the V_o_ ratio in the back-channel was 23.7% and that in the bulk-channel was 32.7%, i.e., there was a decreased V_o_ and increased M–O in the back-channel region compared with the bulk-channel region (Fig. 6(c,d)). Figure 6(e) shows the Hf 4d spectra for the back- and bulk-channel regions of the a-IGZO film with HfO_2_ passivation. The Hf 4d spectra of these films were also studied because the core level binding energy of Ga 3d at 20.6 eV is near that of Hf 4 f (18.9 eV)^22,23,30^.

**Figure 7:**
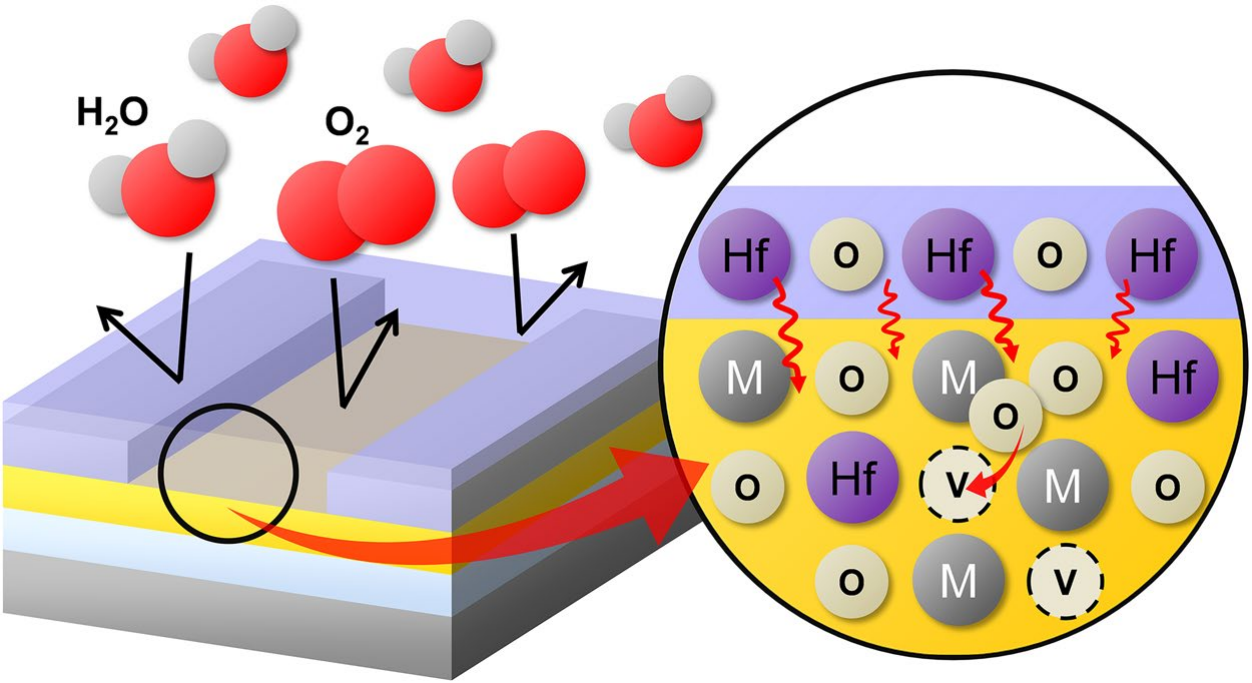
Schematic mechanism for the stability enhancement by solution-processed HfO_2_ passivation layer of a-IGZO TFT.

It has been reported that Hf^4+^ can act as an oxygen binder and reduce V_o_ in AOS films^28,31–34^. This could be due to the low standard electrode potential (SEP) of Hf (−1.70 V), which could strengthen M–O more effectively than Ga (SEP: −0.52 V) in the a-IGZO film^35^. The Hf^4+^ diffused into the back-channel region of the a-IGZO film and reduced the V_o_ concentration in the back-channel layer. The resulting reduced instability of the a-IGZO TFT with HfO_2_ passivation was attributed to the barrier effect of the back surface and a reduction in the number of V_o_-related trap sites (Fig. 7).

In conclusion, a DI water-based solution-processed HfO_2_ passivation layer was successfully prepared at the low temperature of 150 °C. This prevented any interaction between ambient gases and the back surface of an a-IGZO TFT, and the diffusion of Hf^4+^ into the channel layer suppressed oxygen deficiencies. PBS testing for 10,000 s revealed that the bias instability ΔV_th_ improved from 4.83 V for the a-IGZO TFT without passivation to 1.68 V with HfO_2_ passivation. Moreover, the stability enhancement by HfO_2_ passivation was superior to that by PMMA passivation. The DI water-based solution-processed HfO_2_ passivation is competitive with organic passivation from the perspective of a low-temperature process for flexible electronics.

## Methods

### Fabrication of the a-IGZO TFTs

The a-IGZO TFTs were fabricated with an inverted staggered structure. The a-IGZO film (40-nm-thick) was deposited using radio-frequency (RF) magnetron sputtering on a heavily doped p-type Si wafer having a thermally oxidized SiO_2_ coating 1,200 Å thick. The IGZO target was three inches in diameter and consisted of In_2_O_3_:Ga_2_O_3_:ZnO at a ratio of 1:1:1 (mol%). After channel deposition, the samples were annealed in ambient air at 300 °C for 1 h. Aluminum layers (200-nm-thick) were deposited for source/drain electrodes by thermal evaporation using a shadow mask. The width and length of the channel were 1,000 and 150 μm, respectively.

### Fabrication of the passivation layer

To fabricate the HfO_2_ passivation layer, the HfO_2_ precursor solution (0.1 M) was prepared by dissolving hafnium (IV) chloride (HfCl_4_; Aldrich, 98%) in DI water. For the Y_2_O_3_ passivation layer, the Y_2_O_3_ precursor solution was made using yttrium (III) chloride hexahydrate (YCl_3_·6H_2_O; Aldrich, 99.9%) using the same molar ratio and solvent as the HfO_2_ precursor solution. For the PMMA passivation layer, the PMMA precursor solution was synthesized by dissolving 40 mg/mL of PMMA ([CH_2_C(CH_3_)(CO_2_CH_3_)]_n_; Aldrich; M_w_ ca.15,000) in butyl acetate (CH_3_COO(CH_2_)_3_CH_3_; Sigma–Aldrich, 99%). All solutions were stirred for 1 h at room temperature and aged for 24 h. The precursor solutions were then spin-coated onto the fabricated a-IGZO TFTs at 3,000 rpm for 30 s, and annealed at 100 to 250 °C in air for 1 h.

### Electrical characteristics and chemical properties measurement

The electrical characteristics of the a-IGZO TFTs without passivation and with PMMA, Y_2_O_3_, and HfO_2_ passivation were measured using a semiconductor parameter analyzer (model HP 4156 C; Agilent Technologies). To analyze the stability, PBS tests were conducted for 10,000 s with V_GS_ = 20 V and V_DS_ = 10.1 V in air. The thermal decomposition characteristic of the precursor solution was measured using TGA/DSC (model SDT Q600; TA Instruments). The chemical properties of the channel and passivation layer of samples were measured using XPS (model K-Alpha; Thermo Fisher Scientific).

## References

[1] Nomura K (2004). Room-temperature fabrication of transparent flexible thin-film transistors using amorphous oxide semiconductors. Nature.

[2] Kumomi H, Nomura K, Kamiya T, Hosono H (2008). Amorphous oxide channel TFTs. Thin Solid Films.

[3] Hong S, Park JW, Kim HJ, Kim Y, Kim HJ (2016). A review of multi-stacked active-layer structures for solution-processed oxide semiconductor thin-film transistors. J. Inf. Disp..

[4] Choi Y (2010). Carrier-suppressing effect of scandium in InZnO systems for solution-processed thin film transistors. Appl. Phys. Lett..

[5] Xu X, Feng L, He S, Jin Y, Guo X (2012). Solution-processed zinc oxide thin-film transistors with a low-temperature polymer passivation layer. IEEE Electron Device Lett..

[6] Jeong JK, Won Yang H, Jeong JH, Mo Y-G, Kim HD (2008). Origin of threshold voltage instability in indium-gallium-zinc oxide thin film transistors. Appl. Phys. Lett..

[7] Nomura K, Kamiya T, Hosono H (2012). Stability and high-frequency operation of amorphous In–Ga–Zn–O thin-film transistors with various passivation layers. Thin Solid Films.

[8] Dong C (2014). Improvements in passivation effect of amorphous InGaZnO thin film transistors. Mater. Sci. Semicond. Process.

[9] Seo S-J, Yang S, Ko J-H, Bae B-S (2011). Effects of sol-gel organic-inorganic hybrid passivation on stability of solution-processed zinc tin oxide thin film transistors. Electrochem. Solid State Lett..

[10] Nam S (2011). Solvent-free solution processed passivation layer for improved long-term stability of organic field-effect transistors. J. Mater. Chem..

[11] Kim KH, Kim Y-H, Kim HJ, Han J-I, Park SK (2011). Fast and stable solution-processed transparent oxide thin-film transistor circuits. IEEE Electron Device Lett..

[12] Park SK, Kim Y-H, Kim H-S, Han J-I (2009). High performance solution-processed and lithographically patterned zinc–tin oxide thin-film transistors with good operational stability. Electrochem. Solid State Lett..

[13] An S, Mativenga M, Kim Y, Jang J (2014). Improvement of bias-stability in amorphous-indium-gallium-zinc-oxide thin-film transistors by using solution-processed Y_2_O_3_ passivation. Appl. Phys. Lett..

[14] Bukke RN, Avis C, Jang J (2016). Solution-processed amorphous In–Zn–Sn oxide thin-film transistor performance improvement by solution-processed Y_2_O_3_ passivation. IEEE Electron Device Lett..

[15] Choi UH (2016). Electrical stability enhancement of GeInGaO thin-film transistors by solution-processed Li-doped yttrium oxide passivation. J. Phys. D-Appl. Phys..

[16] Kim JH, Rim YS, Kim HJ (2014). Homojunction solution-processed metal oxide thin-film transistors using passivation-induced channel definition. ACS Appl. Mater. Interfaces.

[17] Barraud E, Bégin-Colin S, Le Caër G, Villieras F, Barres O (2006). Thermal decomposition of HfCl_4_ as a function of its hydration state. J. Solid State Chem..

[18] Avis C, Kim YG, Jang J (2012). Solution processed hafnium oxide as a gate insulator for low-voltage oxide thin-film transistors. J. Mater. Chem..

[19] Al-Kuhaili MF, Durrani SMA, Bakhtiari IA, Dastageer MA, Mekki MB (2011). Influence of hydrogen annealing on the properties of hafnium oxide thin films. Mater. Chem. Phys..

[20] de Rouffignac P, Park J-S, Gordon RG (2005). Atomic layer deposition of Y_2_O_3_ thin films from yttrium tris(N,N’-diisopropylacetamidinate) and water. Chem. Mat..

[21] Majumdar D, Chatterjee D (1991). X‐ray photoelectron spectroscopic studies on yttria, zirconia, and yttria‐stabilized zirconia. J. Appl. Phys..

[22] Wang SJ (2003). Reaction of SiO_2_ with hafnium oxide in low oxygen pressure. Appl. Phys. Lett..

[23] Engelhard M, Herman J, Wallace R, Baer D (2011). As-received, ozone cleaned and Ar^+^ sputtered surfaces of hafnium oxide grown by atomic layer deposition and studied by XPS. Surf. Sci. Spectra.

[24] Wei C-Y (2009). Pentacene-based thin-film transistors with a solution-process hafnium oxide insulator. IEEE Electron Device Lett..

[25] McIntyre, N. S. Quantitative surface analysis of materials 83–104 (American Society for Testing and Materials, 1978).

[26] Nayak PK, Hedhili MN, Cha D, Alshareef HN (2013). High performance In_2_O_3_ thin film transistors using chemically derived aluminum oxide dielectric. Appl. Phys. Lett..

[27] Kulchaisit C (2013). Reliability improvement of amorphous InGaZnO thin-film transistors by less hydroxyl-groups siloxane passivation. J. Disp. Technol..

[28] Son D-H (2011). Effect of hafnium addition on the electrical properties of indium zinc oxide thin film transistors. Thin Solid Films.

[29] Park JH (2014). Simple method to enhance positive bias stress stability of In–Ga–Zn–O thin-film transistors using a vertically graded oxygen-vacancy active layer. ACS Appl. Mater. Interfaces.

[30] Carli R, Bianchi CL (1994). XPS analysis of gallium oxides. Appl. Surf. Sci..

[31] Kim C-J (2009). Amorphous hafnium-indium-zinc oxide semiconductor thin film transistors. Appl. Phys. Lett..

[32] Choi YJ, Kim SS, Lee SY (2012). Effect of hafnium addition on Zn-Sn-O thin film transistors fabricated by solution process. Appl. Phys. Lett..

[33] Chong E, Jo KC, Lee SY (2010). High stability of amorphous hafnium-indium-zinc-oxide thin film transistor. Appl. Phys. Lett..

[34] Chong E, Lee SY (2011). Influence of a highly doped buried layer for HfInZnO thin-film transistors. Semicond. Sci. Technol..

[35] Jeong WH (2010). Investigating addition effect of hafnium in InZnO thin film transistors using a solution process. Appl. Phys. Lett..

